# Identifying Recurrence Risk Factors in CT-Confirmed Acute Appendicitis in Adults Managed Non-operatively During the COVID-19 Pandemic

**DOI:** 10.7759/cureus.28794

**Published:** 2022-09-05

**Authors:** Chris B Richards, Laura K Pendower, Pinky D Kotecha, Karl Elmqvist, Fungayi N Chinaka, Ivan Tomasi

**Affiliations:** 1 General Surgery, Guy's and St Thomas' National Health Service (NHS) Foundation Trust, London, GBR; 2 Radiology, Guy's and St Thomas' National Health Service (NHS) Foundation Trust, London, GBR

**Keywords:** survival analysis, ct scan, recurrent appendicitis, non-operative management, acute appendicitis

## Abstract

Background/Objective: Acute appendicitis (AA) is predominantly managed with appendectomy, but can be treated non-operatively, leading to a high risk of recurrence. Non-operative management has been more common since the COVID-19 pandemic affected the feasibility of performing surgery. This case-control study analyzed non-operatively managed patients in order to identify clinical and radiological factors associated with recurrence risk.

Methods: Over 12 months, 48 adults with CT-proven AA managed non-operatively were identified, and followed up for at least six further months to assess them for recurrence (readmission to hospital more than 14 days after discharge and after symptom resolution, requiring treatment for appendicitis). Clinical and CT data were collected and a Cox regression survival analysis was performed to produce hazards ratios (HRs).

Results: Of the 48 patients, 12 (25%) experienced a recurrence up until the end of the follow-up period, eight of whom were then treated operatively, and four treated non-operatively. On the univariate analysis, greater recurrence risk was observed in patients with diabetes mellitus, higher heart rate (on admission and maximum value during admission), lower white cell count and neutrophils and appendiceal wall thinning on CT. On the multivariate analysis, diabetes mellitus (HR=7.72, p=0.021) and higher heart rate (HR=1.08, p=0.018) were associated with statistically significant greater recurrence risk.

Conclusions: Diabetes mellitus and higher heart rate on admission are associated with greater recurrence risk of AA managed non-operatively. No CT findings were associated with statistically significant greater risk. Clinicians should, therefore, consider DM and heart rate when making decisions on appendicitis management, especially during the COVID-19 pandemic but also beyond it.

## Introduction

Acute appendicitis (AA) is the most common surgical emergency, and it has been treated with appendectomy since its first recognized case in 1880 [1]. Advances in surgical techniques and technology have further asserted surgery as the typical management of AA, which is now primarily treated with laparoscopic appendectomy. 

An alternative AA treatment is antibiotic therapy which for uncomplicated AA causes less complications and less sick days compared to surgery [2-3], but leads to a recurrence rate of 27.3% at one year and 39.1% at five years [3]. A 2020 study found that 10 days of antibiotics was non-inferior to surgery for uncomplicated AA according to patient-reported quality of life, but 25% required appendectomy before 90 days [4]. Large meta-analyses recommend that the choice between surgery and antibiotics for uncomplicated AA should be based on consideration of several factors, including patient preference [2, 5-6].

In cases of AA with abscess there is also the option of draining the abscess radiologically and this has been recommended in cases of abscesses greater than 5 cm in diameter [7]. In these cases, there is a recurrence risk as there is with antibiotics alone, as the appendix itself is not removed.

The coronavirus disease 2019 (COVID-19) pandemic has led to the limitation of availability of hospital beds (particularly intensive care), theater space, and theater staff. Furthermore, for patients testing positive for COVID-19 peri-operatively, 30-day mortality is 23.8%, and 51.2% have pulmonary complications resulting in a 30-day mortality of 38.0% in this group [8]. There was also initially a concern regarding COVID-19 transmission to theater staff with laparoscopic surgery, although a recent systematic review concluded that there was no clear evidence for this [9]. These factors led to higher rates of AA treated non-operatively [10], therefore, providing an opportunity to assess the outcomes of a larger pool of patients treated without surgery. Another study analyzed AA cases across 97 centers during the “first wave” of the pandemic, finding that antibiotics alone was successful in 80% of cases at 90-day follow-up and that it resulted in less complications and less financial cost than surgery [10].

Computed tomography is the most sensitive and specific diagnostic investigation of AA [11] and it is also faster, more readily available, and less operator-dependent than ultrasonography (USG). However, it does involve ionizing radiation equivalent to 2.6 years of background radiation in the case of CT of the abdomen and pelvis which is typically performed to assess for AA [12]. Therefore, it is usually avoided, particularly in younger patients, unless USG is unavailable and the clinical diagnosis is uncertain. CT can, however, provide objective measures and has, therefore, been incorporated with clinical measures into the Appendicitis Severity Index (APSI) which accurately predicts complicated AA (perforated or gangrenous) [13]. The measures used are: age over 51 years; fever; symptoms for more than 48 h; and the following on CT: appendiceal diameter ≥14 mm; periappendiceal fluid; extraluminal air; and perityphlitic abscess.

However, there is currently no tool aiming to predict recurrence in AA patients managed non-operatively, meaning with antibiotics alone or interventional radiology drainage (IR-drain) of an abscess. Greater recurrence risk following non-operative management of AA has been shown to be associated with male gender [14] and abscess on CT [15], however, to this date no study has analyzed clinical factors and all radiological signs used in the development of the APSI tool. This study aims to analyze AA patients from a large urban teaching hospital which has been widely recognized as a high-intensity COVID-19 center. This is in order to further identify clinical and radiological factors which are associated with greater recurrence risk in CT-confirmed AA treated non-operatively. Identifying higher recurrence risk in non-operative management would reasonably support the decision to perform appendectomy. However, the high risk of mortality in patients with peri-operative COVID-19 infection [8] necessitates the clinician to be highly selective of patients for surgical management during the pandemic. Therefore, knowledge of recurrence risk might help surgeons worldwide to rationalize the decision to perform surgery or not, both during the pandemic and beyond.

The objectives were to: identify all adults with non-operatively managed CT-proven AA in a 12-month period; identify which patients had a recurrence; and identify associations between clinical and radiological factors and the recurrence risk.

This article was previously presented as an oral presentation at the 2021 World Society of Emergency Surgery Congress on September 10, 2021. This article was also previously posted to the Research Square preprint server on February 27, 2022.

## Materials and methods

All adults diagnosed with AA at our institution from March 1, 2020 to February 28, 2021 were retrospectively identified using the hospital database. Cases which were managed non-operatively were identified and labelled as either managed with antibiotics only or IR-drain. Non-operatively managed patients who underwent a CT scan during their admission but before attempted non-operative management were then identified. For those with multiple admissions, only the initial admission was used in the data analysis. The eligibility criteria were therefore: age over 18 years; diagnosed with AA from March 1, 2020 to February 28, 2021; treated non-operatively; and underwent a CT scan during their admission but before attempted non-operative management (defined as before more than one dose of antibiotics or before IR-drain).

The patients had their demographic, clinical, and biochemical data reviewed to provide data on age, gender, presenting history, past medical history (PMH), vital signs, blood tests, and antibiotic management. Of the continuous variables, there were several for which the data was significantly skewed so they were converted to the following categorical variables: more than two days of pain on admission, antibiotic course longer than seven days, admission longer than two days, C-reactive protein (CRP) >100 mg/L, and temperature >37.5°C (fever). Due to the small number of patients with a history of many comorbidities, several of them were combined into the following groups: cardiovascular risk factors; bowel disease; and hepatobiliary disease.

CT scans were reviewed by a radiologist and assessed for the same signs which were used in the development of the APSI [13]. These were: appendiceal diameter; appendiceal wall thickness; thinning of appendiceal wall; periappendiceal fat stranding and fluid; intraluminal and extraluminal air; cecal wall thickening >3 mm; appendicolith; and periappendiceal abscess. The assessor was blinded to clinical, biochemical, and outcome data.

Patients were followed up until six months after the study period (up to September 2021), meaning their follow-up time varied from approximately six months to 18 months. They were assessed for recurrence using the hospital systems and by telephone in case they had presented to a different hospital. AA recurrence was defined as reattendance to hospital requiring treatment for appendicitis more than 14 days after discharge and after resolution of symptoms. Therefore, the patients were categorized as recurrent, or non-recurrent. Reattendance within 14 days was considered persistent appendicitis rather than recurrence. The time to recurrence was noted for the recurrent group and for the non-recurrent group it was the time to end of follow-up for the purposes of the survival analysis.

The data were then analyzed using IBM SPSS Statistics Version 26.0 (IBM Corp., Armonk, NY). A Cox regression survival analysis was performed to correct for the variation in follow-up times between patients. This compared each variable with time to recurrence, producing recurrence hazard ratios (HRs) and p-values. Statistical significance on the univariate analysis was defined as p-value < 0.20. The significant measures were then included in a multivariate analysis to evaluate their impact on recurrence risk independent of each other, therefore, correcting for any potentially confounding variables. p-value < 0.05 was deemed statistically significant on this multivariate analysis.

Approval from an ethics committee was not deemed necessary as this was a retrospective study where the patient-identifying data has been anonymized. Since the selection of patients and definition of recurrence were based on objective criteria being met, selection and recall bias were minimalized.

## Results

**Figure 1:**
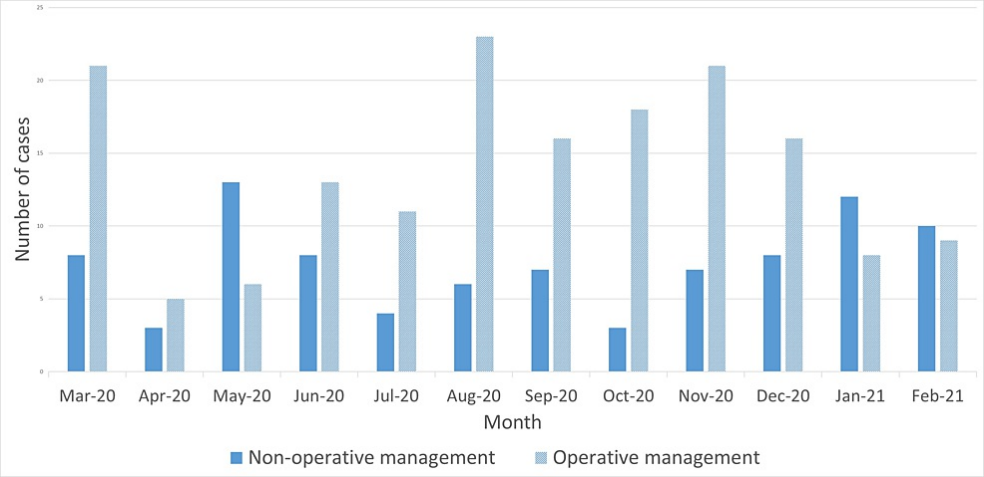
Graph showing the number of cases of AA managed non-operatively and operatively by month. AA, acute appendicitis

There were 280 diagnoses of AA within the one-year period, which occurred in 258 different patients. Of these, 92 (36%) were managed non-operatively on their initial admission. Some 48 of these patients underwent a CT scan during their admission but before attempted non-operative management. Of these 27 were male and 21 were female, and mean average age was 47.5 years (range 24-97 years). Figure 1 shows the management of AA by month at our institution over the 12-month study period.

Of the 48 patients of interest, there were 12 (25%) who had experienced a recurrence up until the end of the follow-up period. Table 1 shows the demographic data for recurrent and non-recurrent groups. Two patients in the non-recurrent group underwent interval appendectomy in the follow-up period.

**Table 1:** Patient demographics and number of patients with each comorbidity in their medical history.

		Total (n=48)	Recurrent (n=12)	Non-recurrent (n=36)
Mean age in years (range)		47.5 (21-97)	46.1 (24-66)	48 (21-97)
Male		27	6	21
Female		21	6	15
Diabetes mellitus		6	3	3
Cardiovascular risk factors	Any	8	3	5
	Hypertension	5	1	4
	Ischemic heart disease	2	0	2
	Obesity	2	1	1
	Chronic kidney disease	3	1	2
Bowel disease	Any	8	2	6
	Inflammatory bowel disease	1	0	1
	Colitis	1	0	1
	Bowel malignancy	2	1	1
	Chronic constipation	2	0	2
	Irritable bowel syndrome	2	1	1
Hepatobiliary disease	Any	5	2	3
	Gallstones	2	1	1
	Cholecystitis	2	0	2
	Portal hypertension	1	1	0
	Liver cirrhosis	1	1	0

Of the included patient there was some missing data for the following variables: appendix diameter (four patients); appendix wall thickness (12 patients); thinning of appendiceal wall (20 patients); periappendiceal fat stranding (one patient); periappendiceal fluid (two patients); appendiceal intraluminal air (four patients); extraluminal air (three patients); cecal wall thickness (seven patients); appendicolith (one patient); use of IV antibiotics (one patient); and length of antibiotic course (five patients).

The recurrence analysis for the clinical factors is shown in Table 2. There was no significant impact of age or gender on recurrence risk. Lower recurrence risk was observed in IR-drain patients compared to those treated with antibiotics alone but this was not significant.

**Table 2:** Recurrence analysis for demographic and clinical factors. Unadjusted HRs refer to the univariate analysis and the adjusted HRs ratios refer to the multivariate analysis. CRP, C-reactive protein; WCC, white cell count; CI, confidence interval; IV, intravenous; IR-drain, interventional radiology drainage *Denotes statistically significant results.

Variable	Unadjusted HR	p-value	Adjusted HR (95% CI)	p-value
Age	0.997	0.852		
Male	ref	n/a		
Female	1.38	0.581		
Antibiotics only	ref	n/a		
IR-drain	0.498	0.508		
>2 days of pain on admission	1.10	0.876		
Admitted for >2 days	1.70	0.367		
Antibiotic course >7 days	1.02	0.975		
IV antibiotics	2.10	0.34		
Diabetes mellitus	2.96	0.105*	7.72 (1.36-43.7)	0.021*
Bowel disease	1.10	0.907		
Hepatobiliary disease	2.39	0.271		
Cardiovascular risk factors	1.63	0.466		
Maximum CRP>100	1.72	0.357		
Admission CRP>100	0.984	0.978		
Febrile anytime during admission	0.989	0.987		
Febrile on admission	0.354	0.321		
Maximum WCC	0.927	0.386		
Admission WCC	0.86	0.104*		
Maximum neutrophils	0.879	0.215		
Admission neutrophils	0.821	0.075*	0.842 (0.621-1.14)	0.267
Maximum heart rate	1.03	0.094*		
Admission heart rate	1.05	0.017*	1.08 (1.01-1.47)	0.018*

Greater recurrence risk was observed in patients with diabetes mellitus (DM) (unadjusted HR=2.96, p=0.105) and this was significant so included in the multivariate analysis. Bowel disease and hepatobiliary disease increased recurrence risk but this was not statistically significant. Maximum C-reactive protein (CRP) > 100 mg/L was associated with greater recurrence risk but admission CRP > 100 mg/L was associated with lower recurrence risk. Higher admission white cell count (WCC) (unadjusted HR=0.860, p=0.104) and admission neutrophils (unadjusted HR=0.821, p=0.075) were associated with lower recurrence risk and were statistically significant. Maximum heart rate during admission (unadjusted HR=1.03, p=0.094) and heart rate on admission (unadjusted HR=1.05, p=0.017) were both associated with statistically significantly greater recurrence risk.

Table 3 shows the recurrence analysis for the CT data. Thinning of appendiceal wall on CT (unadjusted HR=2.67, p=0.129) was associated with significantly greater recurrence risk. However, none of the other radiological outcome measures were statistically significant. Fat stranding was present in all 47 patients in which it could be assessed for.

**Table 3:** Recurrence analysis for the radiological factors on CT. Unadjusted HRs refer to the univariate analysis and the adjusted HRs refer to the multivariate analysis HR, hazards ratio; CI, confidence interval *Denotes statistically significant results

Variable	Unadjusted HR	p-value	Adjusted HR (95% CI)	p-value
Appendix diameter (mm)	1.13	0.377		
Appendiceal wall thickness (mm)	1.37	0.411		
Thin appendiceal wall	2.67	0.129*	1.86 (0.319-10.9)	0.489
Periappendiceal fat stranding	n/a	n/a		
Periappendiceal fluid	1	1		
Intraluminal air	0.565	0.589		
Extraluminal air	0.037	0.349		
Cecal wall thickness >3 mm	0.475	0.22		
Appendicolith	1.17	0.799		
Abscess	0.452	0.309		

The multivariate analysis is also shown in Tables 2-3. Admission WCC and maximum heart rate were not included in the analysis due to their strong association with other more significant variables (admission neutrophils and admission heart rate, respectively). When adjusted for the impact of the other variables DM was shown to be associated with an even greater recurrence risk and this was statistically significant (adjusted HR=7.72, p=0.021). This also applies to admission heart rate (adjusted HR=1.08, p=0.018). The other variables were shown to not be significant on the multivariate analysis.

## Discussion

This study provides a valuable contribution to the existing literature regarding recurrent AA following non-operative management, by identifying further risk factors. Previous studies have found greater recurrence risk in patients of male gender [14], and those with abscess on CT [15], but this study has identified greater heart rate on admission and DM as significant factors to be considered by the clinician. This is the first study to analyze the relationship of a large number of radiological factors as well as clinical factors, with AA recurrence risk, in large urban teaching hospital. A large Taiwanese study found that AA recurrence risk was greater in males, those under 18 years old, and those treated with a percutaneous drain [16]. However, it included children and did not analyze any CT data. This study has assessed the impact on recurrence of all the CT findings used to develop the APSI [13], whereas other studies which involved CT findings did not incorporate as many of them [14-15, 17]. Multiple CT factors have been incorporated in one study previously which found that risk was greater in patients with an appendicolith [17], but the authors did not differentiate between recurrent AA and persistent AA, which is an important distinction. Another strength of this study is that the participants were followed up until between six and 18 months after their initial admission, with a large meta-analysis showing that average time to AA recurrence varies from 3.4 to 7.0 months [2]. 

In this study higher heart rate on admission was associated with significantly greater recurrence risk on the multivariate analysis. Heart rate is not currently used in tools to predict complicated AA [13] or diagnose AA [18], possibly due to concerns around it being caused by fever, pain or anxiety. However, it has been shown to predict perforated AA [19], and this study adds to the evidence that a higher heart rate should be treated with caution when deciding on AA management. DM was also associated with greater recurrence risk on multivariate analysis, and therefore it may be appropriate to manage diabetic patients surgically in order to prevent an AA recurrence. This is in keeping with another study which has shown that diabetic women have a greater recurrence risk than non-diabetic women [20], likely due to poorer wound healing in diabetics. Gender did not impact recurrence risk in this study so was not included in the multivariate analysis.

Recurrence risk was greater in those admitted for more than two days, those treated with antibiotics for more than seven days, and those treated with IV antibiotics, but these results are likely to have been confounded by other factors affecting the management decisions regarding length of admission and antibiotic therapy.

The CRP value >100 g/L at any time during the admission was associated with greater recurrence risk but notably CRP >100 mg/L on admission was not. This is in line with the delayed rise in CRP or “lag” which is known to be associated with inflammation generally, and also highlights that admission to hospital to monitor CRP during non-operative management may be beneficial, in order to not be falsely reassured by a lower CRP on admission. Higher WCC was not associated with a greater recurrence risk which is in keeping with the APSI study which found that WCC did not predict complicated AA [13]. High WCC is a well-documented feature in AA, and incorporated into sensitive and specific diagnostic scoring tools [18]. However, in this study high WCC was associated with a lower recurrence risk, as was presence of fever although this result was not statistically significant. Histologically-proven complicated AA has been associated with high CRP and high procalcitonin levels independently [21]. A systematic review of biomarkers in AA found that procalcitonin and interleukin-6 levels were predictive for perforated appendix but procalcitonin lacked sensitivity and both were high in cost [22]. Therefore, these biomarkers are unlikely to provide affordable insight into AA recurrence risk.

Thinning of the appendiceal wall was associated with greater recurrence risk but none of the other CT findings were. AA is primarily a clinical diagnosis but imaging plays a vital role in cases of diagnostic uncertainty, with USG being considered the first-line modality [23]. This study provides little evidence to suggest that performing more CTs would be valuable in order to predict recurrence, especially given the radiation exposure involved. However, in cases where a CT has been performed a thin appendiceal wall should alert the clinician that recurrence risk may be greater. Abscess on CT has been shown to be associated with greater recurrence risk [15] and CT findings are used to predict complicated AA [13], so it is likely that CT could play a role in prediction of recurrence that would warrant further investigation. CT has also been shown to be useful pre-operatively, markedly reducing the negative appendectomy rate, defined as the pathologically normal appendix removed from the patient for suspected appendicitis. Other studies have shown that this reduction is as much as from 22% to 7% [24], and from 13% to 5% [25]. These figures might indicate that 5%-7% of patients in this study may have been misdiagnosed as AA on CT.

Figure 1 shows that a greater proportion of cases of AA were managed non-operatively during May 2020 and January to February 2021. Inpatients in the UK testing positive for COVID-19 peaked during April 2020 and January 2021 [26] so the trend of non-operative management highlights a greater preparedness of this surgical department for the so-called “second wave” of the pandemic. This is compared with the “first wave” when the high mortality associated with operating on COVID-19 patients was not yet well published [8]. Of the 12 patients who experienced a recurrence, eight of them were treated surgically for their second episode, and the other four were treated with antibiotics alone. It remains to be seen whether there will be repeated COVID-19 waves requiring more cases of AA to be managed non-operatively in the future. If this is the case, this would enhance the relevance of the findings presented here which aid the surgeon in deciding which cases would require surgery at initial presentation, and which should be treated non-operatively before being offered interval appendectomy.

Limitations of this study include that it is retrospective, and that statistical significance on the univariate analysis was defined as p < 0.20 due to a relatively small sample size. The associations presented here, particularly on the multivariate analysis, could be further evaluated in a larger study either over a greater time period or across multiple centers. It is likely that at least 50 patients in both recurrent and non-recurrent groups would be required for the training data to design a model to reliably predict recurrent AA. There was also no power calculation in this study and it has been suggested that at least five events (recurrent cases) per predictor variable are needed for problems to be uncommon when using a Cox regression model [27]. Larger sample size would also allow more robust analysis of the comorbidities that were grouped together due to small prevalence in our population, such as hypertension and obesity. Therefore, a larger multicenter study is planned to investigate further the risk factors of AA recurrence over a longer follow-up period.

It could also be considered a limitation that both uncomplicated and complicated AA were included in the study, however, this is in keeping with other studies investigating recurrence risk factors including the presence of perforation [14], abscess [15], and appendicolith [16]. This study is, therefore, a valuable addition to this literature, particularly for the on-call surgeon making a decision on whether to operate where CT imaging, which could determine the presence of complicated appendicitis, may not be appropriate or available. 

## Conclusions

Acute appendicitis is a variable and multi-faceted disease with many factors affecting its severity, making its course relatively difficult to predict. It is, therefore, challenging to confidently decide whether certain patients would be reasonably treated non-operatively. This highlights the importance of involving patients in discussions regarding their management decisions. The work presented here has shown an association of greater AA recurrence risk with DM and higher heart rate on admission, which will aid the clinician in being highly selective of patients for surgical management despite the risks during the pandemic, such as high mortality if infected with COVID-19. Caution should also be taken in patients with a thinned appendiceal wall on CT, although this study provides little evidence to suggest that a CT should be performed to assess for risk of recurrence in the future. This is the first study to analyze multiple clinical and CT findings in a highly diverse AA population, and therefore provides valuable findings to aid on-call emergency surgeons working in similar centers across the globe. 

## References

[1] Meljnikov I, Radojcić B, Grebeldinger S, Radojcić N (2009). [History of surgical treatment of appendicitis]. Med Pregl.

[2] Sallinen V, Akl EA, You JJ (2016). Meta-analysis of antibiotics versus appendicectomy for non-perforated acute appendicitis. Br J Surg.

[3] Salminen P, Tuominen R, Paajanen H (2018). Five-year follow-up of antibiotic therapy for uncomplicated acute appendicitis in the APPAC randomized clinical trial. JAMA.

[4] Flum DR, Davidson GH, Monsell SE (2020). A randomized trial comparing antibiotics with appendectomy for appendicitis. N Engl J Med.

[5] Podda M, Cillara N, Di Saverio S (2017). Antibiotics-first strategy for uncomplicated acute appendicitis in adults is associated with increased rates of peritonitis at surgery. A systematic review with meta-analysis of randomized controlled trials comparing appendectomy and non-operative management with antibiotics. Surgeon.

[6] Yang Z, Sun F, Ai S, Wang J, Guan W, Liu S (2019). Meta-analysis of studies comparing conservative treatment with antibiotics and appendectomy for acute appendicitis in the adult. BMC Surg.

[7] Forsyth J, Lasithiotakis K, Peter M (2017). The evolving management of the appendix mass in the era of laparoscopy and interventional radiology. Surgeon.

[8] Archer JE, Odeh A, Ereidge S, Salem HK, Jones GP, Gardner A (2020). Mortality and pulmonary complications in patients undergoing surgery with perioperative SARS-CoV-2 infection: an international cohort study. Lancet.

[9] Antunes D, Lami M, Chukwudi A (2021). COVID-19 infection risk by open and laparoscopic surgical smoke: a systematic review of the literature. Surgeon.

[10] Javanmard-Emamghissi H, Hollyman M, Boyd-Carson H (2021). Antibiotics as first-line alternative to appendicectomy in adult appendicitis: 90-day follow-up from a prospective, multicentre cohort study. Br J Surg.

[11] Shogilev DJ, Duus N, Odom SR, Shapiro NI (2014). Diagnosing appendicitis: evidence-based review of the diagnostic approach in 2014. West J Emerg Med.

[12] Mettler FA Jr, Mahesh M, Bhargavan-Chatfield M (2020). Patient exposure from radiologic and nuclear medicine procedures in the United States: procedure volume and effective dose for the period 2006-2016. Radiology.

[13] Avanesov M, Wiese NJ, Karul M (2018). Diagnostic prediction of complicated appendicitis by combined clinical and radiological appendicitis severity index (APSI). Eur Radiol.

[14] Lien WC, Lee WC, Wang HP, Chen YC, Liu KL, Chen CJ (2011). Male gender is a risk factor for recurrent appendicitis following nonoperative treatment. World J Surg.

[15] Felber A, Catalano D, Stafford C (2019). What is the long-term follow-up of nonoperatively treated patients with appendicitis?. Am Surg.

[16] Liang TJ, Liu SI, Tsai CY, Kang CH, Huang WC, Chang HT, Chen IS (2016). Analysis of recurrence management in patients who underwent nonsurgical treatment for acute appendicitis. Medicine (Baltimore).

[17] Shindoh J, Niwa H, Kawai K (2010). Predictive factors for negative outcomes in initial non-operative management of suspected appendicitis. J Gastrointest Surg.

[18] Frountzas M, Stergios K, Kopsini D, Schizas D, Kontzoglou K, Toutouzas K (2018). Alvarado or RIPASA score for diagnosis of acute appendicitis? A meta-analysis of randomized trials. Int J Surg.

[19] Kearney D, Cahill RA, O'Brien E, Kirwan WO, Redmond HP (2008). Influence of delays on perforation risk in adults with acute appendicitis. Dis Colon Rectum.

[20] Tsai MC, Lin HC, Lee CZ (2017). Diabetes increases the risk of an appendectomy in patients with antibiotic treatment of noncomplicated appendicitis. Am J Surg.

[21] Li Y, Zhang Z, Cheang I, Li X (2020). Procalcitonin as an excellent differential marker between uncomplicated and complicated acute appendicitis in adult patients. Eur J Trauma Emerg Surg.

[22] Acharya A, Markar SR, Ni M, Hanna GB (2017). Biomarkers of acute appendicitis: systematic review and cost-benefit trade-off analysis. Surg Endosc.

[23] Debnath J, George RA, Ravikumar R (2017). Imaging in acute appendicitis: what, when, and why?. Med J Armed Forces India.

[24] Chan J, Fan KS, Mak TLA (2020). Pre-operative imaging can reduce negative appendectomy rate in acute appendicitis. Ulster Med J.

[25] Webb EM, Nguyen A, Wang ZJ, Stengel JW, Westphalen AC, Coakley FV (2011). The negative appendectomy rate: who benefits from preoperative CT?. Am J Roentgenol.

[26] (2021). Gov.uk [Internet]. UK Health Security Agency [updated]. https://coronavirus.data.gov.uk/details/healthcare.

[27] Vittinghoff E, McCulloch CE (2007). Relaxing the rule of ten events per variable in logistic and Cox regression. Am J Epidemiol.

